# Study on Vacuum Breakdown Properties of Surface-Modified 304 Stainless Steel Electrodes Based on Fractal Theory

**DOI:** 10.3390/nano15050340

**Published:** 2025-02-22

**Authors:** Shiqing Wang, Shenming Zhao, Bo Liu, Weihong Shan, Hao Wei, Dayan Ma, Hongbo Wang

**Affiliations:** 1 State Key Laboratory for Mechanical Behavior of Materials, Xi’an Jiaotong University, Xi’an 710049, China; 2 National Key Laboratory of Electrical Materials and Electrical Insulation, Xi’an Jiaotong University, Xi’an 710049, China; 3 National Laboratory of Intense Pulsed Radiation Simulation and Effect, Northwest Institute of Nuclear Technology, Xi’an 710024, China; 4 School of Materials Science and Engineering, Chang’an University, Xi’an 710061, China

**Keywords:** modified stainless steel, fractal dimension, breakdown threshold, surface morphology

## Abstract

This paper reports on the effect of the micro-morphological characteristics of stainless steel electrodes on vacuum breakdown properties under the action of a strong electric field generated by high-power electric pulses. Using chemical passivation modification and atomic layer deposition (ALD) technology, alumina composite films were prepared on the surface of the stainless steel electrodes to reshape the surface microstructure of the electrodes. The surface morphology features of the electrodes were characterized in detail. Based on fractal theory, a fractal model based on the box dimensional method was proposed to quantitatively describe the morphological and structural characteristics of the film, and its relationship with the vacuum breakdown properties was established. The results indicated that the fractal dimension effectively reflected the complexity of the electrode morphology and could serve as a key parameter to evaluate the vacuum breakdown performance of the electrodes, which showed a negative correlation with the change tendency of the electrode breakdown threshold.

## 1. Introduction

Vacuum electronic devices such as pulsed power drive sources and high-power microwave sources are widely used in various fields such as radar systems, communication technologies, defense applications, and medical devices. However, explosive electron emission from metal surfaces in strong electromagnetic fields is the primary reason for vacuum breakdown [1,2]. Suppressing the electrical breakdown phenomenon is one of the key issues to enhance the performance and the reliability of these advanced types of equipment [3,4,5,6]. Among the many factors that lead to the breakdown of metals in a vacuum, i.e., the surface morphology of the electrodes, gap size, geometric shape, electrode structure, and material properties, the microscopic surface structural properties, such as cracks, surface micro-bumps, and inclusions, play a key role in determining the breakdown threshold because of surface-controlled explosive electron emissions [7,8,9,10].

In recent years, the influence of the surface structure of metal electrodes on the vacuum breakdown properties has received widespread attention. C.S. Mayberry et al. [11] coated TiO_2_, SiO_2_, Cu, Al, and other materials on the surface of stainless steel to change its mesoscopic structure. Their results showed that after magnetron sputtering of those materials with a film thicknesses of 500 nm and above, the in situ-formed micro-protrusions on the electrode surface could improve the electrode surface quality, and resulted in the enhancement of the strong field breakdown threshold, but the enhancement effect was limited. Yu Zhang et al. [12] improved the surface structure of titanium plate electrodes by mechanical polishing and chemical polishing, respectively. Their results showed that when the roughness of the electrode was reduced from 3.5 μm to 0.35 μm, the breakdown threshold was improved by 35%. However, when the roughness was small enough to reach the nm level, the surface roughness was no longer the main factor affecting the breakdown threshold. In addition, roughness was only sensitive to changes in the top valleys of the surface, and it could not provide enough information to characterize the complexity/irregularity of the surface morphology [7]. Tao Zhang et al. [13] prepared alumina samples with different sizes and density of pores to investigate the relationship between the pore distribution and the breakdown strength. Their results showed that large pore defects had a significant effect on the dielectric breakdown performance of alumina ceramics, while small pores had little effect. Moreover, there were different shapes and irregular distribution of pores, so the structure obtained from the analysis still had limitations. Due to the complex structure and information of electrode surface morphology, the quantitative parameters obtained by the traditional characterization methods are affected by the measurement scale and the resolution of the instrument. A comprehensive description of the electrode surface features still lacks systematic research. Therefore, it is extremely important to select a suitable method for the complete quantitative description of complex surface morphology. Therefore, the introduction of the fractal approach for the complete quantitative description of complex surface morphology is considered.

Since the establishment of fractal theory by Mandelbrot in 1975 [14], fractal geometry has become a powerful tool for quantifying complex phenomena [15,16], which also provides a new way to study irregular surfaces. Different fractal and multifractal techniques can be used to characterize the surface microforms and their effects on various physical, chemical, and mechanical properties [17,18,19]. Being based on the self-similarity of surfaces at different scales, the fractal dimension is insensitive to structural details. The structure can be characterized by a single descriptor fractal dimension (D), which can provide information about the complexity of different surface topographies [20]. The quantitative study of the relationship between the fractal dimension and the vacuum breakdown properties of metal electrode surfaces is still rare.

In this study, through chemical passivation modification and atomic layer deposition (ALD) technology, alumina composite films were prepared on the surface of the stainless steel metal electrodes to reshape the microstructure of the electrodes. A fractal model based on the box dimensional method was proposed to quantitatively describe the morphological and structural characteristics of the films, and its relationship with the vacuum breakdown properties was established. This work would provide useful routes for the performance prediction and optimization of the pulse power drive and high-power microwave sources with a high vacuum breakdown threshold.

## 2. Experimental Details

### 2.1. Materials and Preparation

Two types of 304 stainless steel disc substrates with diameters of 20 mm and 200 mm (Deli Machinery Hardware Co., Ltd., Shenzhen, China), respectively, were used in this work. Stainless steel discs with a diameter of 20 mm were used to study the morphological characteristics, while the larger substrates were utilized as electrodes to assess the breakdown performances. The stainless steel substrates were firstly polished sequentially using sandpaper (Golden Sun E-commerce Co., Ltd., Dongguan, China) ranging from 200# to 1200#. Then they were cleaned with deionized water and dried with cold air. Subsequently, the pretreated samples were modified in a nitric acid pickling passivation solution, with passivation times of 30 min, 50 min, and 70 min, respectively. The different pickling times were chosen with the aim of systematically changing the surface morphology of the samples, thus allowing us to study the effect of these morphological changes in the subsequent fractal dimension and breakdown properties. The detailed processing steps were as follows: water washing→ ultrasonic degreasing with cleaning agent → water washing → pickling → water washing → passivation → water washing → neutralization treatment → water washing → drying. Among them, pickling passivation solution comprised a mixture of HNO_3_ (analytical grade, Sigma-Aldrich, St. Louis, MO, USA) and HCl (analytical grade, Sinopharm, Beijing, China). The detailed composition of the solution is provided in Table 1. The cleaning agent contained 1% NaOH, 0.5% Na_2_SiO_3_, and 0.2% sodium dodecylbenzene sulfonate in alkaline solution (analytical grade, Alfa Aesar, Ward Hill, MA, USA). Deionized water was used throughout the experiments, with a resistivity of at least 18 MΩ·cm. The drying was carried out for 15 min in a drying oven at 50 °C.

Al_2_O_3_ thin films were deposited on chemically passivated 304 stainless steel substrates by ALD. Silicon substrates (size: 20 × 20 mm; thickness: 525 ± 15 μm; resistivity: 0.01–0.015 Ω·cm; Jingxin Electronic Technology Co., Ltd., Quzhou, China) were placed into the deposition chamber simultaneously to check the film thicknesses of the samples. Before deposition, the stainless steel and silicon wafer substrates were sequentially cleaned with acetone and alcohol for 10 min each to remove surface impurities. Trimethylaluminum (Purity: 99.999%; Aimuyuan Scientific Equipment Co., Ltd., Nanjing, China) served as the aluminum source precursor, while hydrogen peroxide (an aqueous solution of H_2_O_2_ with a mass fraction of 24% to 35%; Si-nopharm Group Chemical Reagent Co., Ltd., Shijiazhuang, China) was employed as the oxygen source. High-purity nitrogen gas was used as the purge gas in each cycle. The detailed experimental parameters are presented in Table 2.

### 2.2. Characterization Equipment

Scanning electron microscopy (FE-SEM, Hitachi SU8230, Hitachi Scientific Instruments Co., Ltd., Beijing, China, voltage: 15 kV, resolution: 0.6 nm) was employed to characterize the morphology and thickness of the films. The chemical composition was analyzed by energy dispersive spectroscopy (EDS, Hitachi SU8230, Hitachi Scientific Instruments Co., Ltd., Beijing, China, Voltage: 15 kV). Surface morphology and depth profiles of the stainless steel microstructures were estimated by an ultra-high-resolution laser confocal microscope (Leica TCSSP8 STED 3X, Wetzlar, Germany). ImageJ (version 1.53, Wayne Rasband, National Institutes of Health, Bethesda, MD, USA) and Nano Measurer software (version 1.2) were utilized to analyze the surface images of the film layers obtained from SEM for the calculation of the size, distribution, and coverage of cracks and lamellar structures. The fractal dimension of the images was primarily calculated by MATLAB software (version: 9.13.0 (R2022b), The MathWorks Inc., Natick, MA, USA).

### 2.3. Breakdown Characteristic Test

**Figure 1:**
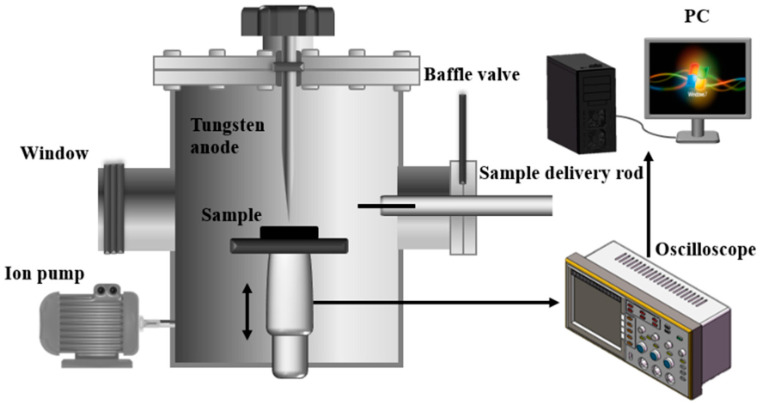
Schematic diagram of the breakdown test device.

The schematic diagram of the modified stainless steel electrode strong field pulsed breakdown characteristic testing device is shown in Figure 1. The device consists of a discharge chamber and a sample chamber. During the pulsed breakdown tests, the vacuum level of the discharge chamber was maintained below 8 × 10^−4^ Pa. After charging the capacitor to the specified voltage (U) using the fixed-voltage method, the charging circuit switch was turned off, and the sample stage was controlled by a stepper motor to gradually shorten the gap distance between the cathode and the anode until breakdown occurred. The stepper motor was stopped and the cathode-anode distance (d) at this point was recorded, thereby calculating the breakdown threshold (E). And the same stepping speed needed to be used in the experiments; generally, 0.2 mm·min^−1^ was used in the experiments. The breakdown threshold was calculated by the following formula:

(1) $$E=\frac{U}{d}$$

In Equation (1), E was the breakdown threshold (unit: kV/mm) calculated from the above equation, U represented the specified voltage (unit: kV), and d represented the electrode gap distance (unit: mm) measured when breakdown occurred. In addition, in order to minimize the error of the experimental results, it is necessary to carry out a number of repetitive tests to take the average value.

### 2.4. Box-Counting Method

Secondary electron scanning electron microscope (SEM) is a powerful surface morphology analysis technique. In-depth morphological features can be derived from the SEM grayscale images by appropriate technical processing. The surface morphology of the samples influences the reflection intensity of the secondary electrons, resulting in variations in image brightness and darkness, or grayscale differences. Consequently, the brightness intensity distribution of the SEM image of a three-dimensional modified surface directly reflects its true morphological properties, allowing for the calculation of fractal dimension directly from SEM images.

**Figure 2:**
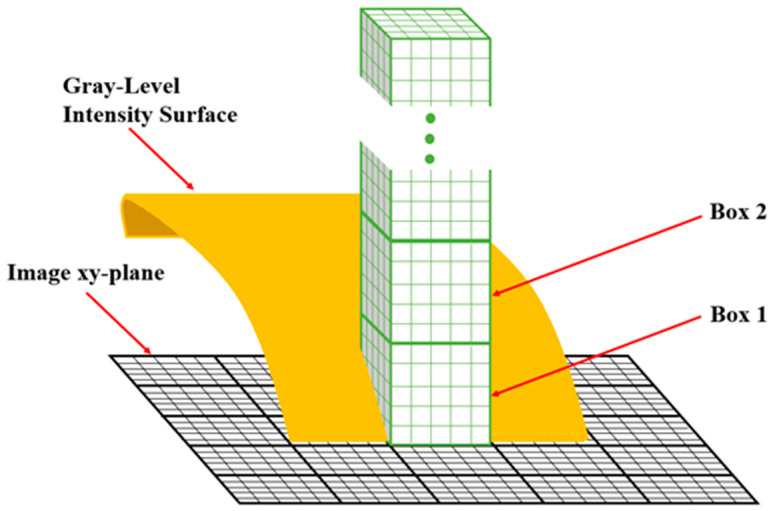
Schematic diagram of calculating fractal dimension by box dimension method.

In a typical process for the calculation of fractal dimension, an SEM image is input into a computer, and the typical process of calculating the fractal dimension, the SEM image is first input into the computer and the image is processed using a program written in MATLAB, which starts with preprocessing using a median filter to reduce impulse noise and smoothing the image using a Gaussian filter, followed by converting the image to binary format using adaptive thresholding. Subsequently, the box dimension method is employed to calculate the surface fractal dimension. According to the principles of the box dimension method [21,22], the M × M grayscale image is transformed into a three-dimensional image I_M×M_ mapped onto a 3D plane, as illustrated in Figure 2. In this representation, the x, y, and z coordinates correspond to the length and width of image I, and the intensity value of I, respectively. The xy plane is divided into non-overlapping grids of size s × s pixels, where s is an integer varying from smin = 2 to smax = M/2. s’ is the height of the box, given by s’ = s × G/M, where G is the total number of gray levels in the image. U_s_ and B_s_ are the maximum and minimum gray levels within the region, respectively. The number of grids that contain at least one gray level between U_s_ and B_s_ is denoted as N, which varies with different values of s. r is the ratio of the grid width s to the image side length M, i.e., s/M. M is a constant, and the grid size serves as the scale for measuring the gray levels of the image. Therefore, the relationship between s and N can be used to calculate the fractal dimension of the image gray data.

Based on the definition of measure dimension, the fractal dimension D of set R in three dimensions can be defined as:

(2) D=log⁡N/log⁡(1/r)

Here, N represents the measurement result when measuring R with r. Different measurements with different rulers can yield different results, from which the fractal dimension D can be calculated. Therefore, the fractal dimension of the image grayscale data can be derived by using the relationship between s and N from formula (2):

(3) $$N={\left(s/M\right)}^{-D}$$

That is:

(4) log⁡N=−Dlog⁡s+C

In the formula, D represents the box dimension of the image and is a constant. By calculating different N values for different s values, a double logarithmic plot of s and N can be created, and the slope of the fitted line using the least squares method can yield −D, which can then be used to calculate the fractal dimension.

## 3. Result and Discussion

### 3.1. Morphological Characteristics of the Electrode Surface After Different Modification Treatments

**Figure 3:**
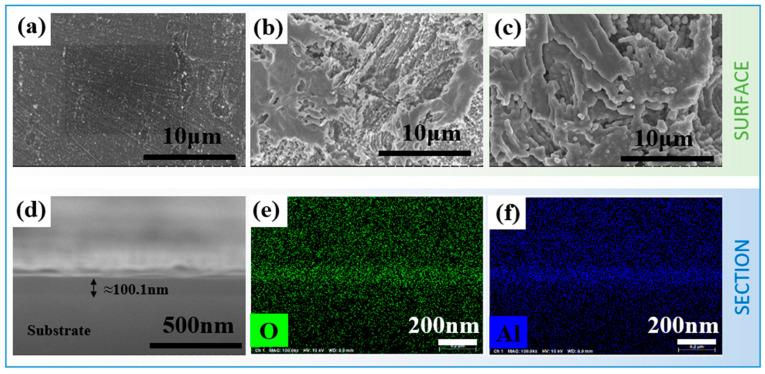
SEM surface morphology of stainless steel before and after modification: (**a**) pretreated 304 stainless steel substrate, (**b**) nitric acid passivation, (**c**) the nitric-acid-passivated sample after the deposition of Al_2_O_3_ film by ALD, (**d**) cross-sectional SEM image of the ALD-coated sample, (**e**) cross-sectional EDS map of element O of the ALD-coated sample, (**f**) cross-sectional EDS map of element Al of the ALD-coated sample.

Figure 3 shows typical SEM images of the stainless steel before and after modification. It can be indicated from Figure 3a that the surface of the untreated sample is relatively flat, exhibiting only minor scratches that originated from the pretreatment grinding process. In contrast, the surface morphology of the passivated sample shown in Figure 3b becomes inhomogeneous, which can be attributed to the localized chemical dissolution of the metal during the passivation process. The surface structure is arranged in a reticulated pattern decorated with grooves and holes, which results from the dynamic reorganization of the passivated film. Figure 3c shows the SEM of the nitric-acid-passivated sample after ALD deposition of alumina film. It can be observed that the overall morphology does not change significantly after ALD coating, indicating the excellent uniformity and conformality of ALD technology. High-resolution cross-sectional SEM observation (Figure 3d) also confirms the high uniform and conform feature of the ALD film. The film thickness of all the samples deposited by ALD is about 100 nm, which is insufficient to alter the surface morphology of the sample. Detailed quantitative analyses of the morphological features, including information on mean size and density, are provided in subsequent paragraphs and summarized in Table 3.

**Figure 4:**
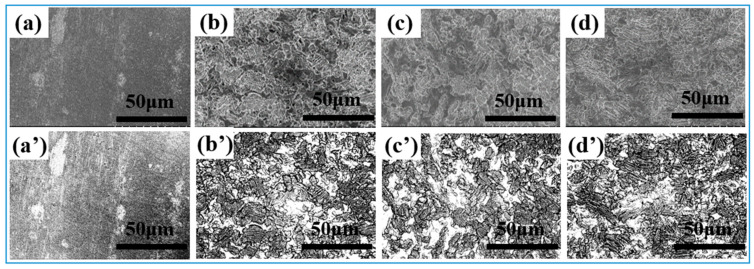
SEM and grayscale images of nitric acid passivation sample after processing: (**a**,**a′**) 304-S, (**b**,**b′**) 30-N, (**c**,**c′**) 50-N, (**d**,**d′**) 70-N.

Figure 4a–d show the SEM morphology of the samples after pickling for different times. The above images were processed using ImageJ software to extract the morphological information by applying inverse thresholding. The specific threshold values were determined based on the grayscale intensity distribution of the images so that the sample features and background could be clearly distinguished. We set the lower threshold at 50 and the upper threshold at 200 on an 8-bit grayscale (0–255). These values were selected after a series of preliminary tests to ensure that all the relevant features of the samples were accurately captured without including background noise. As shown in Figure 4a′–d′, the topographic images after grayscale processing show clear brightness contrasts, clearly presenting the surface structure features. In the anti-thresholding processed image of the sample after pickling and passivation, the bright area represents the continuously covered film, and the black area represents the bare surface after passivation treatment. The surfaces of the samples are accompanied by dissolution, exfoliation, and dynamic reorganization, and generate new oxide particles that are aggregated to form a uniform and continuous island-like structure. With the prolongation of the passivation time, the size of the island structure becomes smaller, while the density increases.

**Figure 5:**
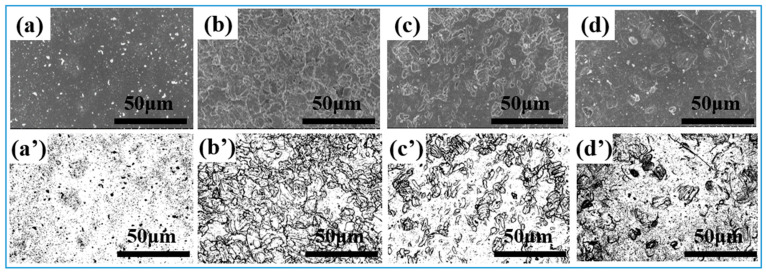
SEM and grayscale images of the nitric acid passivation samples after the deposition of Al_2_O_3_ film by ALD (**a**,**a′**) 304-SA, (**b**,**b′**) 30-NA, (**c**,**c′**) 50-NA, (**d**,**d′**) 70-NA.

Figure 5 shows the SEM morphology and processed images of the atomic layer deposition modified samples pretreated by different passivation times. The results reveal that after the ALD modification, Al_2_O_3_ particles aggregate on the surface of the stainless steel, resulting in an ellipsoid-like geometric feature. In comparison with the original stainless steel substrate, the roughness of the ALD-modified stainless steel surface is reduced, and the defects such as voids and oxide channels produced by pickling are diminished. This indicates that ALD can enhance the integrity of the stainless steel surface.

A binary image with only black and bright areas is obtained by ImageJ software processing. The black area contains island-like structures, small lamellae, and particles within the island-like structures, while the bright area indicates the continuous film layers. By using Nano Measurer software, the coverage, average size, maximum, and minimum size values of the structures sizes are statistically analyzed. Table 3 presents the calculated results. As can be seen, the size and the overall coverage of the continuous island-shaped areas are highly dependent on the duration time of the pickling process, which initially increases but then decreases with the prolongation of the pickling time. Consistent with previous analyses, the small lamellae in the interior of the island structures originate from the acid pickling passivation procedure owing to the corrosion of the grain boundaries and the matrix dissolution. As the passivation time increases, the differentiation of this lamellar structure becomes more pronounced. After 70 min of passivation, the lamellar structure disintegrates into fine particles with a diameter of approximately 2.12 μm. After the ALD coating, numerous alumina nanoparticles form on the surface with sizes ranging from 30 to 50 nm, while the distance between the nanoparticle surfaces is mainly concentrated between 80–100 nm. After the ALD coating process, the surface of the sample becomes smoother, flatter, denser, and more uniform. However, the size of the island structure and the reticulated inner layer remain nearly unchanged, reflecting the conformal nature of ALD [23]. Based on the two groups of samples, it can be seen that the size changes in the island structure and the inner layer are consistent with the area coverage, and the larger the size is, the higher the area coverage is.

**Figure 6:**
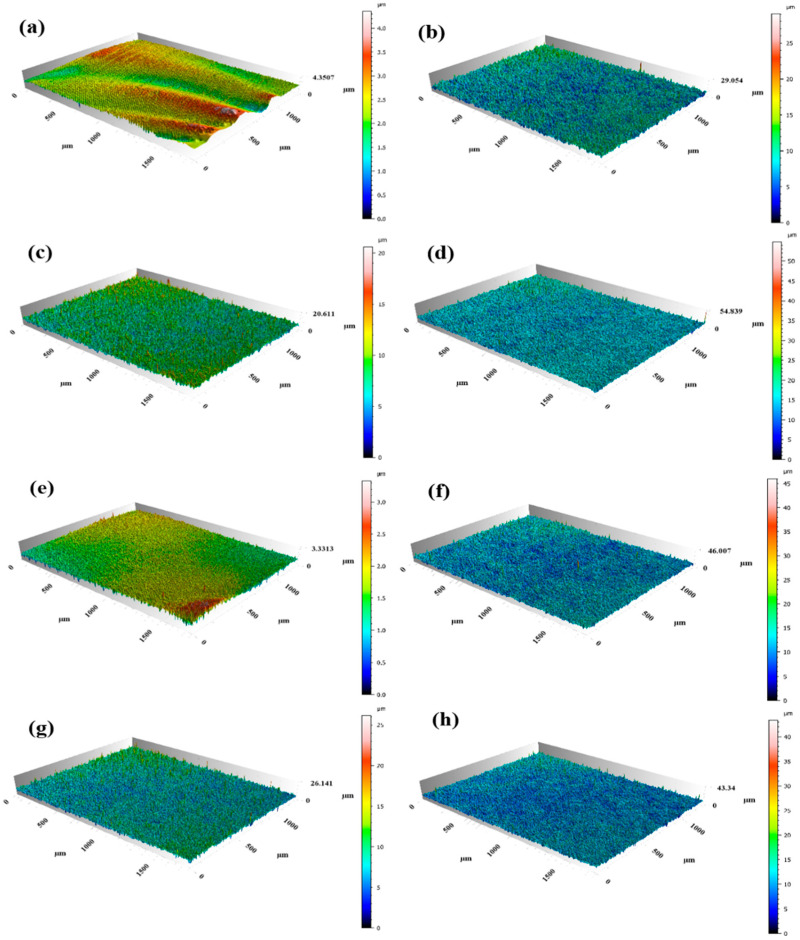
Laser confocal test results of different samples: (**a**) 304-S, (**b**) 30-N, (**c**) 50-N, (**d**) 70-N, (**e**) 304-SA, (**f**) 30-NA, (**g**) 50-NA, (**h**) 50-NA.

**Figure 7:**
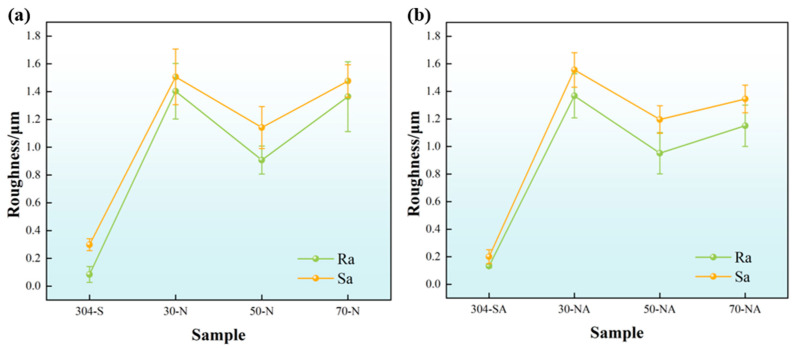
Line roughness and surface roughness of different samples: (**a**) pickling passivation, (**b**) ALD-deposited Al_2_O_3_.

The 3D morphology images of the modified samples are shown in Figure 6. Figure 7 shows the results of the line roughness R_a_ (Arithmetic Average Roughness) and surface roughness S_a_ (Arithmetic Mean Roughness) measured by laser confocal microscopy of the different samples. As can be seen, the surface roughness R_a_ of the original 304 stainless steel is 0.103 μm, with evenly distributed scratches on the surface. After the pickling and passivation procedure, the scratches on the surface are eliminated. When the pickling time is extended to 70 min, the surface roughness increases significantly. The surface roughness of the 304 stainless steel after modification by different processes is shown in Figure 7. As can be seen from Figure 7a, the surface roughness of the 304 stainless steel after pickling and passivation is much larger than that of the original substrate, due to the gradual destruction of the original oxide layer by corrosion. The roughness of the pickling passivated samples is first reduced and then increased with the increase in pickling passivation time. The reduction tendency of roughness can be attributed to the dissolving of the intrinsic oxide layer and the formation of the chemical conversion film. While the increase in the roughness should be ascribed to prolonging the pickling time, which induces the rupture of the newly formed passivation layer. The roughness of the ALD-coated (Figure 7b) samples decreases slightly compared with that of the nitric-acid-passivated samples, but the overall roughness variation tendency is similar.

### 3.2. Fractal Dimension of Electrode Surface After Different Modification Treatments

**Figure 8:**
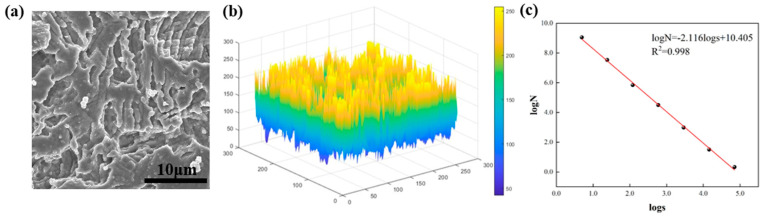
Fractal dimension calculation example: (**a**) SEM of the 70-NA sample, (**b**) extracted grayscale surface, (**c**) fractal dimension fitting calculation.

The accuracy of fractal dimension analysis can be affected by the resolution of the data; lower resolutions may lead to an underestimation of the surface complexity, while higher resolutions may introduce noise that affects the results. Furthermore, fractal dimension calculations are sensitive to the scale range used in the analysis, and if the scale range is not chosen properly, the true fractal behavior of the surface may not be captured. To address these limitations, we combined fractal dimension analysis with SEM images with high resolution and carefully selected the scale range based on preliminary analysis. Figure 8 shows the calculation process of the fractal dimensions. Taking the 70-NA sample as an example, the SEM image of the sample with the size of 256 × 256 × 8 bit is given, and the grayscale surface information extraction and binarization are processed by MATLAB programming software. The extracted gray scale surface represents the gray value distribution of the SEM image, which is used to characterize the surface morphology of the modified stainless steel. The grayscale intensity in the SEM image is converted into a three-dimensional surface, where the x and y axes correspond to the spatial pixel coordinates (unit: pixels, size: 256 × 256), and the z axis represents the grayscale intensity (unit: dimensionless, ranging from 0 to 255) which correlates with the relative height variations of the surface morphology. And finally, the fractal dimension of this image can be calculated. To ensure the accuracy and consistency of the results, the process was repeated several times on different images and the average of the fractal dimensions was taken after the calculation. As can be seen in Figure 8c, a logs–logN plot of s and N is created, and the fitted result for the fractal dimension D is determined as 2.116 and the calculated linear correlation coefficient R^2^ = 0.998, indicating the good correlation of the logs–logN, and the image has good fractal characteristics in the range of computational scales. Those results prove that it is feasible to use the fractal dimension to quantitatively describe the morphological characteristics of the modified surface of stainless steel.

**Figure 9:**
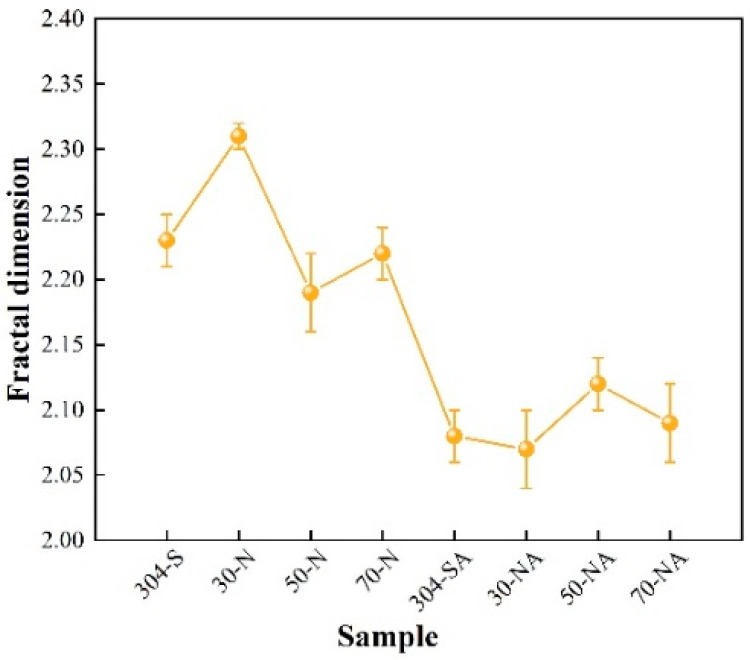
Fractal dimension of different samples.

The value of the fractal dimension (D) indicates the degree of irregularity, self-similarity, and spatial filling of the surface. A larger D value corresponds to a more complex shape and rich microstructural details of the surface. It can be seen from Figure 9 that the fractal dimensions of the samples after the ALD alumina coating are smaller than those of the nitric acid pickling passivation samples. Especially, the samples of 50-N and 30-NA show the smallest fractal dimensions because of the relatively simple surface morphology. It can be seen that the variation in the fractal dimension is inconsistent with the surface roughness, but it is basically consistent with the island structural feature, internal lamellar structure size, and film coverage results shown above.

### 3.3. Relationship Between Fractal Dimension and Surface Morphology of Electrodes After Modification Treatment

**Figure 10:**
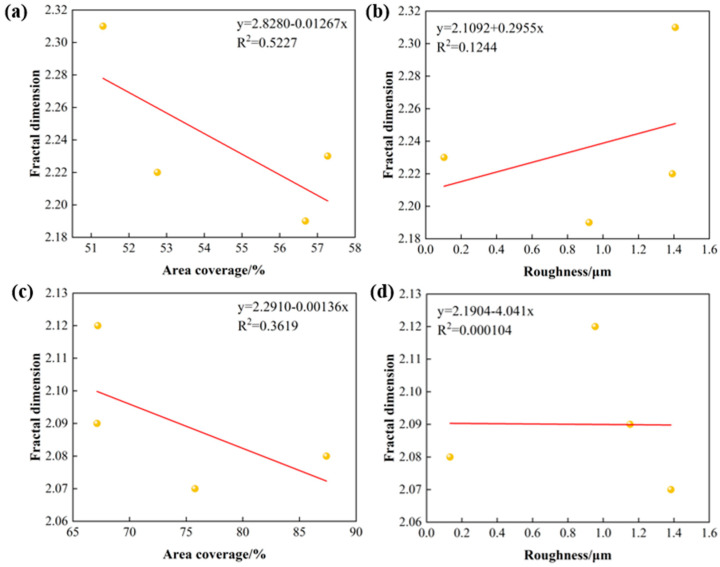
Relationship between fractal dimension and surface topography (**a**) vs. area coverage of pickled passivated samples, (**b**) vs. roughness of pickled passivated samples, (**c**) vs. area coverage of ALD samples, (**d**) vs. roughness of ALD samples.

The relationships between the area coverage, surface roughness, and fractal dimensions of the modified samples are shown in Figure 10. The linear correlation coefficients R^2^ of the area coverage and fractal dimension of the stainless steel surface after chemical passivation and ALD modification are 0.5227, and 0.3619, respectively. The coefficients R^2^ of the fitted curves are negative, indicating the negative correlation between the fractal dimension and area coverage. The linear correlation coefficients R^2^ of the roughness and the fractal dimension are 0.1244 and 0.000104, respectively, which are extremely small, indicating the low correlation between the fractal dimension and roughness. It can be seen that the influencing factors in the horizontal direction after the stainless steel modification, such as the uniformity and integrity of the film coverage, play a dominant role in the surface topographic features, while the influencing factors in the depth direction, such as surface pits and cracks, show a small degree of influence on the surface topographic features. Therefore, it can be assumed that the higher the uniformity and coverage of the film layer on the surface of the stainless steel modified electrode, the lower the complexity of the surface morphology and the smaller the fractal dimension.

### 3.4. Relationship Between Fractal Dimension and Breakdown Threshold of Modified Electrodes

**Figure 11:**
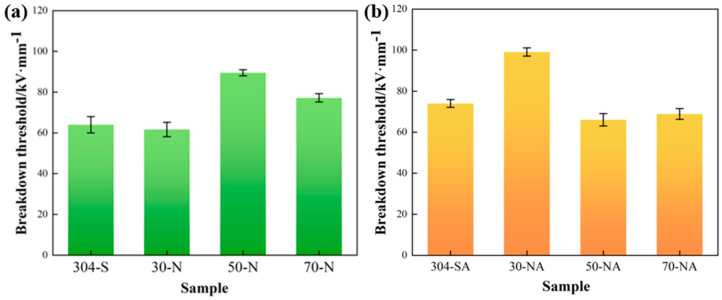
Breakdown threshold of stainless steel electrode modified by (**a**) acid pickling passivation, (**b**) ALD deposition of Al_2_O_3_.

The vacuum breakdown threshold of the samples is shown in Figure 11. The results proved that pickling passivation treatment can effectively improve the breakdown threshold of stainless steel electrodes. Especially, the sample passivated for 50 min exhibits the highest breakdown threshold up to 89.6 kV/mm, and the breakdown threshold is further improved to 99.3 kV/mm after ALD deposition. The sequential enhancement of the breakdown performance can be attributed to the improvement in insulating properties and the reduction in electron emission probability. Previous studies have shown that metal electrode surface structures characterized by high roughness and tip bumps enhance field emission and lower the breakdown threshold [12]. Our study shows that taking advantage of uniform treatment, deep removing the sharp wedges and grooves, and effective reduction in microscopic bumps on the electrode surface by pickling passivation, the field enhancement factor of the electrodes is effectively reduced, and the breakdown strength of the electrodes in the vacuum gap is enhanced remarkably, which is in agreement with what has been described in the previous literature. However, when the pickling passivation time exceeds 50 min, the electrode surface is over-acid-pickling-passivated, which leads to the destruction of the uniform structures, and the newly formed bumps will lead to greatly enhanced field emissions, and therefore the electrode breakdown resistance is reduced. The nitric-acid-passivated 30 min sample after the deposition of Al_2_O_3_ film by ALD has the maximum breakdown threshold. This is because Al_2_O_3_ film has excellent insulating properties [24], and Al_2_O_3_ film closes the defects on the surface of the electrode after passivation, allowing this high-performance dielectric film to become a physical barrier to the movement and collision of electrons, effectively reducing the flashover voltage scattering and hindering the occurrence of vacuum breakdown. However, when the electrode is over-passivated, the density of the agglomerated particles that can act as electron emission sites increases due to the amplification effect of ALD deposition [25]. These particles are usually the typical electrically weak points for surface electric field emission, where the breakdown is usually initiated.

**Figure 12:**
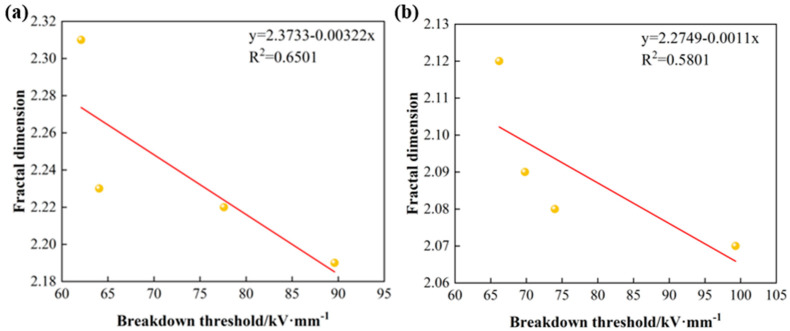
Fractal dimension versus breakdown threshold: (**a**) acid pickling passivation, (**b**) ALD deposition of Al_2_O_3_.

The relationship between the fractal dimensions and the modified samples is shown in Figure 12. The linear correlation coefficients R^2^ of the breakdown threshold and fractal dimension D of the surface-passivated and ALD-modified stainless steel are 0.6501 and 0.5801, respectively. The coefficients of the fitted curves are negative, indicating a negative correlation. This is because of the positive correlation between the film coverage and the breakdown threshold, and the negative correlation between the fractal dimension and the film coverage.

## 4. Conclusions

In order to solve the breakdown failure problem of stainless steel electrodes under high-power pulsed field service conditions, the substrate was chemically passivated and subsequently modified by an alumina thin film via the atomic layer deposition technique. The modification process of stainless steel electrodes was designed and optimized so as to improve its breakdown characteristics. The relationship between the morphological characteristics of the film layer and the breakdown threshold was intensively studied, leading to the following conclusions:

(1) By using the box method, the fractal dimensions were calculated. It was found that there is a strong linear correlation between lnN(r_i_) and ln(1/r_i_), which indicated that the surface morphology of the modified stainless steel exhibited fractal characteristics, and the fractal dimension (D) could be used as an index to characterize the surface features.

(2) After nitric acid pickling and passivation at 60 °C for 50 min, the breakdown threshold of the stainless steel electrode was increased by 39% to 89.6 kV/mm. After the further deposition of 100 nm-thick alumina film by the ALD method, the breakdown threshold of pickling passivated stainless steel for 30 min was increased to 99.3 kV/mm.

(3) The fractal dimension could be used to characterize the surface morphological properties and breakdown performance of the modified stainless steel electrode. The lower the complexity of the surface morphology, the smaller the fractal dimension of the modified electrode surface and the higher the breakdown threshold.

## Figures and Tables

**Table 1:** Sample parameters of nitric acid passivation and ALD-deposited alumina films.

Sample	Pickling Passivation			ALD Cycle/Time
	Solution	Temperature/°C	Time/min	
304-S	-	-	-	-
30-N	10%HNO_3_ + 5%HCl	60	30	-
50-N	10%HNO_3_ + 5%HCl	60	50	-
70-N	10%HNO_3_ + 5%HCl	60	70	-
304-SA	-	-	-	2000
30-NA	10%HNO_3_ + 5%HCl	60	30	2000
50-NA	10%HNO_3_ + 5%HCl	60	50	2000
70-NA	10%HNO_3_ + 5%HCl	60	70	2000

**Table 2:** Preparation parameters of ALD-deposited alumina films.

Composition	Precursor	Precursor Temperature/°C	Oxygen Source	Deposition Temperature/°C	t_1_~t_4_/Time
Al_2_O_3_	TMA	200	H_2_O_2_	25	0.5-17-0.1-16

**Table 3:** Extraction and analysis of morphological characteristics of different samples.

Sample	Size of Small Lamellae Within the Island Structure/μm			Continuous Island Size/μm			Covered Area/%
	Average	Maximum	Minimum	Average	Maximum	Minimum	
304-S	(Bulges) 2.61	4.00	1.28	-	-	-	57.273
30-N	5.03	7.74	2.08	2.87	3.72	2.01	51.314
50-N	7.90	14.35	4.59	12.21	23.81	3.92	56.683
70-N	(Granulate) 2.12	7.01	0.4	9.57	21.59	4.31	52.755
304-SA	(Granulate) 0.7	1.54	0.3	-	-	-	87.379
30-NA	7.62	13.27	4.01	30.72	44.11	17.6	75.777
50-NA	6.47	11.65	3.31	10.2	20.72	4.28	67.203
70-NA	(Granulate) 1.48	3.56	0.73	Continuous island	-	-	67.131

## Data Availability

Data are available upon reasonable request from the authors.
